# Impact of Ketamine on Learning and Memory Function, Neuronal Apoptosis and Its Potential Association with miR-214 and PTEN in Adolescent Rats

**DOI:** 10.1371/journal.pone.0099855

**Published:** 2014-06-10

**Authors:** Ji Wang, Min Zhou, Xiaobin Wang, Xiaoling Yang, Maohua Wang, Chunxiang Zhang, Shuzhi Zhou, Ni Tang

**Affiliations:** 1 Department of Anesthesiology, Affiliated Hospital of Luzhou Medical College, Luzhou, Sichuan Province, China; 2 Department of Pharmacology, Rush Medical College, Rush University, Chicago, Illinois, United Stated of America; University of Memphis, United States of America

## Abstract

Ketamine, an N-methyl-D-aspartate (NMDA) receptor antagonist, is used as a general pediatric anesthetic and anti-depressive drug. Recent studies suggest that ketamine enhances neuronal apoptosis in developing rats. The goal of this study is to explore whether ketamine could result in learning and memory impairment and neurodegeneration in adolescent rats, and if so, whether the effects of ketamine are associated with miR-214 and PTEN expression. Fifty-day-old SD rats were randomly divided into three groups receiving ketamine at 30, or 80 mg/kg, i.p. or saline for seven consecutive days. Twenty-four hours after the last treatment, learning and memory function were tested by the Morris water maze. The rats were then decapitated, and the brains were isolated for detection of neuronal apoptosis and protein PTEN expression by TUNEL and immunohistochemistry respectively. Expression levels of the miR-214 and PTEN in the hippocampus were measured by qRT-PCR and western blot analysis respectively. Ketamine administered to the adolescent rats at a dose of 80 mg/kg rather than the lower dose of 30 mg/kg caused learning and memory impairment, increased the number of apoptotic cells in the hippocampal CA_1_ region, cerebral cortex and subcortical region, decreased the miR-214 levels and increased PTEN protein expression in hippocampus. The results suggest that ketamine at a dose of 80 mg/kg in the adolescent rats is able to induce the learning and memory impairment and neurodegeneration, in which the down-regulation of miR-214 and high expression of PTEN protein may be involved.

## Introduction

Ketamine is a common agent used in pediatric anesthesia. It is well established that ketamine acts primarily through the blockade of N-methyl-D-aspartate(NMDA)-type glutamate receptors to produce a dose-related state of unconsciousness and analgesia for painful procedures [1]. Recently, both animal and clinical studies have demonstrated that ketamine exerts a quick and persistent antidepressant-like effect [2]–[7]. However, the therapeutic role of ketamine in adolescent patients with depressive disorders is still unclear. Ikonomidou reported in 1999 that the repeated administration of ketamine could increase the neuroapoptosis in the developing brain of P7 rats [8]. Since then, an increasing number of studies have demonstrated that ketamine may have the potential to induce the neurodegeneration and cognitive deficits during early development both in vitro and in vivo [9]–[11]. These side effects reported call for caution with the use of ketamine in neonatal, pediatric anesthesia and the long-term anti-depressive therapy. Obviously, more studies in the research field are needed to evaluate the beneficial and the side effects of ketamine, as well as the mechanisms involved. Up to date, the effects of ketamine on learning and memory function, neuronal apoptosis and the mechanisms involved in adolescent rats are still unclear.

MicroRNAs (miRNAs) are a class of endogenous, small, non-coding RNAs and serve as negative regulators for their target mRNAs via promoting degradation of mRNAs or inhibiting their translation. miRNAs are reported to be involved in almost all major cellular functions including cell growth and apoptosis [12], [13]. microRNA-214 (miR-214) may target the phosphatase and tensin homolog deleted on chromosome ten (PTEN)based on bioinformatics analysis and some researches have reported that miR-214 could negatively regulate the PTEN expression by binding to its 3-UTR [14]. In addition, recent studies suggest that the high expression of PTEN could increase tumor cell apoptosis [15]–[21]. The purpose of this study is to explore the potential effects of ketamine on learning and memory function, neuronal apoptosis and the expression of miR-214 and PTEN in adolescent rats.

## Methods

### Subjects

Ninety Male Sprague Dawley (SD) rats (50-day-old) with body weights ranging from 150 to 200 g were obtained from Luzhou Medical College (Luzhou City, Sichuan Province, China) breeding colony. They were housed at 5 per cage with food and water available ad libitum and were maintained on a 12-h light/dark cycle (lights on at 7:00 AM). All experimental procedures involving animals were performed in accordance with the NIH Guide for the Care and Use of Laboratory Animals and the Chinese Society for Neuroscience and Behavior recommendations for animal care. Experimental surgeries and treatments were approved by the ethics committee of the Affiliate Hospital of Luzhou Medical College.

### Procedure

Ninety rats were randomly divided into the following three groups: normal saline (Control), ketamine1 (K_1_) and ketamine 2 (K_2_) group. All treatments in different groups were administered intraperitoneally (i.p.) in a volume of 1 ml/100 g in a blind fashion. Rats in Control group received an i.p. injection of 0.9% normal saline; rats in K_1_ group received an i.p. injection of 30 mg/kg ketamine (Gutian pharmaceutical Co., Fujian, China); rats in K_2_ group received an i.p. injection of 80 mg/kg ketamine. The injection was performed once a day for 7 consecutive days. At 24 h after the last treatment, the 30 rats in each group were again randomly assigned into three sub groups. 10 rats in the sub group 1 received open-field test, then were perfused with 4% paraformaldehyde and decapitated under deep anesthesia, and the detection of neuronal apoptosis and PTEN protein expression levels in hippocampus, cortex and subcortical regions were measured by TUNEL and immunohistochemistry respectively (n = 6). 10 rats in the sub group 2 received Morris water maze test for the analysis of learning and memory function. 10 rats in the sub group 3 were decapitated under deep anesthesia, then expression levels of the miR-214 and PTEN protein in the hippocampus were measured by qRT-PCR and western blot analysis respectively (n = 6).

### Open-field test

In order to assess any possible effects of drug treatments on the spontaneous locomotor activity, rat spontaneous activity analysis was performed in an open field apparatus, which is an arena 45×60 cm surrounded by 50 cm high walls made of brown plywood with a frontal glass wall. The floor of the open field was divided into 9 rectangles (15×20 cm each) by black lines. Animals were gently placed on the left rear quadrant to explore the arena for 5 min. The number of horizontal (crossings) and vertical (rearing) activity performed by each rat during the 5 min observation period was counted by three expert observers in a blinded manner. The horizontal score: if the all of the four feet entered in one grid, score 1 point. The vertical score: when the forelimb left the ground, score 1 point. Experiments were performed between 8 and 10a.m.

### Morris water maze test

The Morris water maze (Taimeng Bioinstrumentation Ltd, Chengdu, China) test was used to determine the changes in learning and memory abilities of the rat. In brief, the water maze was a black circular pool with 120 cm in diameter and 50 cm in height, and was filled with water to a depth of 32 cm at 25°C. A circular transparent plexiglas platform, 8 cm in diameter, was permanently placed in the middle of the northeast quadrant, 40 cm into the pool, and 0.5 cm below the water surface. The maze was placed in a semi-dark room with white walls and follow distal visual extra maze cues present. A black curtain covered the entrance of the room, located at the south wall. Two lamps were located on this wall on each side of accord the entrance and 60 cm above the edge of the pool. One lamp was switched on and the other was off. On the opposite wall (north), two lamps where placed in the corresponding positions, both were switched on. In the middle of the wall was a black dot, 20 cm in diameter, 35 cm above the pool. The eastern wall had a black cross (length 20 cm) in the middle, located at the 35 cm above the pool. A black curtain covered the middle of the western wall. A white dot, 20 cm in diameter, was located on one side of the curtain, at 40 cm above the maze. The investigators and the equipment were located in an adjacent room. The study started on the first day at 24 h after the last injection, and the trial sessions for place navigation within the Morris water maze were continued for a total of 5 days. Each session included 2 searches for the platform from different starting positions of the three quadrants (not including the target quadrant). The sequential order of the searches for the platform was randomly selected from day to day, but all rats at each day had the same order. The performance of the rats was monitored with an overhead video camera connected to an image analyzer (Taimeng Bioinstrumentation Ltd, Chengdu, China) and analyzed by the water maze software HVS WATER 2020. Experiments were performed between 8 a.m. and 2 p.m.

The time to reach the platform was recorded in each trial, with a maximal time limit of 120 s. If the platform was not found within the set time, the computer stopped tracking and recorded the time as 120 s. And if the rat found the platform within 120 s, it was allowed to stay on it for 30 s. Otherwise, the rat was gently guided to find the platform by the experimenter and allowed to remain on the platform for 30 s, and the latency was recorded at 2 min. The time to reach the platform (latency to find the platform) and the length of the swim path (swim distance) were measured with a computerized tracking system. At the end of the training period, the rats were tested on a spatial probe trial in which the platform was removed, and they were allowed to swim freely for 2 min. The time spent to reach the platform initially, ratio of time spent in the target quadrant and the times of crossing the platform were recorded.

### TUNEL method for neural apoptosis in rat hippocampal tissues

Twenty-four hours after the final injection, the rats were deeply anesthetized with sodium pentobarbital and perfused with 4% paraformaldehyde through the left cardiac ventricle and ascending aorta. Brains were harvested and immersed in formalin. After 24 h of fixation in formalin, the samples were embedded in paraffin using standard histological procedures. The slices were done consecutively in hippocampal dentate gyrus, with a thickness of 4 uM. TUNEL staining was performed using an in situ apoptosis detection kit (Roche Applied Science, Mannheim, Germany) according to the manufacturer's instructions. The number of TUNEL-positive cells (Red) was counted under a fluorescence microscope. Unbiased sampling of brain sections was performed by randomly selecting five viewing fields/per section of each brain. To determine the degree of neurodegeneration in a given brain region, a PC-based Image Analysis System (MCID[Microcomputer maging Device], Imaging Research, Inc., St Catherines, Ontario, Canada) interfaced to an Olympus Vanox microscope via a solid state video camera was used for image analysis. The viewing fields (photographs) were counted by a trained expert and later confirmed by two readers blinded to the treatment.

### Immunohistochemistry of PTEN

Briefly, tissue sections were incubated with 0.3% hydrogen peroxide and methanol for 10 minutes and later boiled in 10 mM citrate buffer (pH 6.0) in a 1000 W microwave oven for 10 minutes. Tissue sections were then incubated with the primary antibodies overnight at 4°C. Primary antibodies included rabbit polyclonal antibody to PTEN from Santa Cruz Biotechnology (Santa Cruz, CA, USA). The antibodies were used at dilutions of 1: 50 for PTEN. Then, the sections were incubated with two-step immunohistochemistry detection reagent (PV6001 and PV6002; Zhongshan Goldenbridge, Beijing, China) at 37°C for 30 minutes. A brown color appeared in the slices after 3, 39-diaminobenzidine colorization. As a negative control, some sections were incubated without primary antibody and processed as described above. Sections were counterstained with hematoxylin.

### Western blot analysis of PTEN expression in rat hippocampal tissues

Protein extracted from rat hippocampus from the various groups were used to determine the PTEN protein expression using anti-PTEN antibody (Sigma-Aldrich Corp., USA). Anti-GAPDH antibody (Sigma-Aldrich Corp., USA) was used as control for the Western blot analysis. Hippocampal tissues lysate was extracted using RIPA lysis buffer. Supernatants were collected after centrifugation at 12,000 g for 10 min at 4°C, and protein concentration was quantified by UV spectrophotometry. Protein samples were stored at −70°C before use. Thirty micrograms of total protein from each sample was used to perform SDS-PAGE analysis (12% separating gel and 5% stacking gel). Proteins from the SDS gel were transferred (with 40 mA constant current, 16 V maximal voltage, transferred for 70 min) onto PVDF membranes and blocked for 1 hour. Rabbit anti-mouse PTEN monoclonal antibody at a 1∶1000 dilution was used to incubate the PVDF membrane in a sealed bag overnight at 4°C. After 3 washes for 5 min each in a 0.05% TBS-Tween-20 solution, the PVDF membrane was further incubated in 1∶1000 diluted goat anti-rabbit-HRP antibodies. Membranes were washed in 0.05% TBS-Tween-20 3 times for 5 min each, followed by washing in TBS for 5 min. A3, 3′-diaminobenzidine (DAB) solution was used to visualize the protein blot and the image was scanned with a gel imaging system. An alpha imager analyzer (Alpha Innotech Corporation, San Leandro, CA) was used to measure the optical density (OD) values of the protein bands and quantify the values.

### Hippocampal RNA extraction and qRT-PCR analysis of miRNA-214

Rat hippocampal miRNA-214 expression was determined using quantitative reverse transcriptase-polymerase chain reaction (qRT-PCR) based upon the mirVana qRT-PCR miRNA kit manufacturer's instructions (Ambion Life technologies, USA). cDNA template generated from 50 ng of total RNA was used for qRT-PCR. The miRNA-214 amplification and detection were performed using the Bio-Rad iCycler iQ 5 fluorescence PCR system (Bio-Rad, USA). U_6_ RNA was used as an internal reference for template normalization. The primer (5′-ACAGCAGGCACAGACAGGCAG-3′) for miRNA-214 was purchased from Ambion Life technologies, USA. The fluorescence signal was normalized to unify the internal reference level, and the threshold cycle (C_T_) was set in the exponential amplification phase of the PCR. The relative gene expression of miRNA-214 was calculated based on the number of PCR cycles of miRNA-214. In order to determine the corresponding ΔC_T_ value, the C_T_ value of the target gene miRNA-214 was subtracted from the U_6_ C_T_ value so as to normalize the values. Among the different experimental treatments, the relative expression levels of miRNA-214 were calculated by using the following formula: relative gene expression  = 2^−(ΔC^_T_^ of experimental sample – ΔC^_T_^ of control sample)^.

### Statistical analysis

SPSS 19.0 statistical software was used. All data are expressed as mean ± S.E.M (standard error of the mean). All the experiments were repeated independently at least three times except for the behavioral experiments. For relative gene expression, the mean value of vehicle control group is defined as 100% or 1. The data in qRT-PCR analysis, western blot analysis, TUNEL and immunohistochemistry assay were analyzed by a one-way ANOVA. For the Morris water maze test, the data were analyzed using repeated-measures analysis of variance (RMANOVA). Post-hoc individual means comparisons were conducted by Dunnett's test. All statistical assessments used a significance level of p<0.05.

## Results

### Open-field test

**Figure 1:**
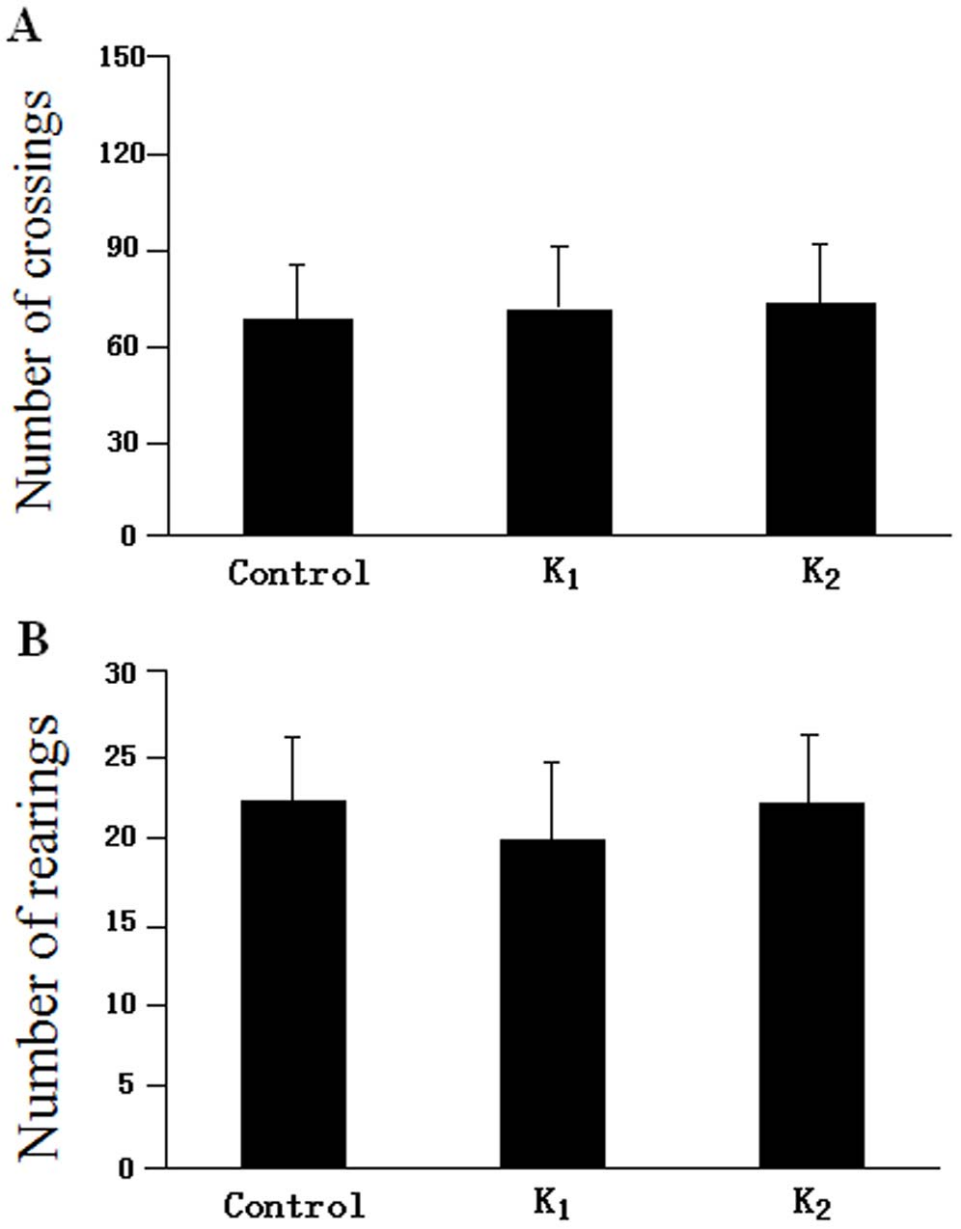
Open field experiment score±S.E.M in Control, K_1_ and K_2_ group. The total scores have no difference in Control, K_1_ and K_2_ group (P>0.05).

Twenty-four hours after the last treatment, 30 rats (n = 10, each group) were trained in the open field test to evaluate spontaneous locomotor activity ability. As shown in Fig. 1, compared with the control group, none of the intraperitoneal (i.p.) injections with ketamine (30 or 80 mg/kg) increase the horizontal [F_(2,27)_ = 0.052, p = 0.812] and vertical scores [F_(2,27)_ = 0.305, p = 0.328] of rats.

### Learning and memory ability by the Morris water maze test

**Figure 2:**
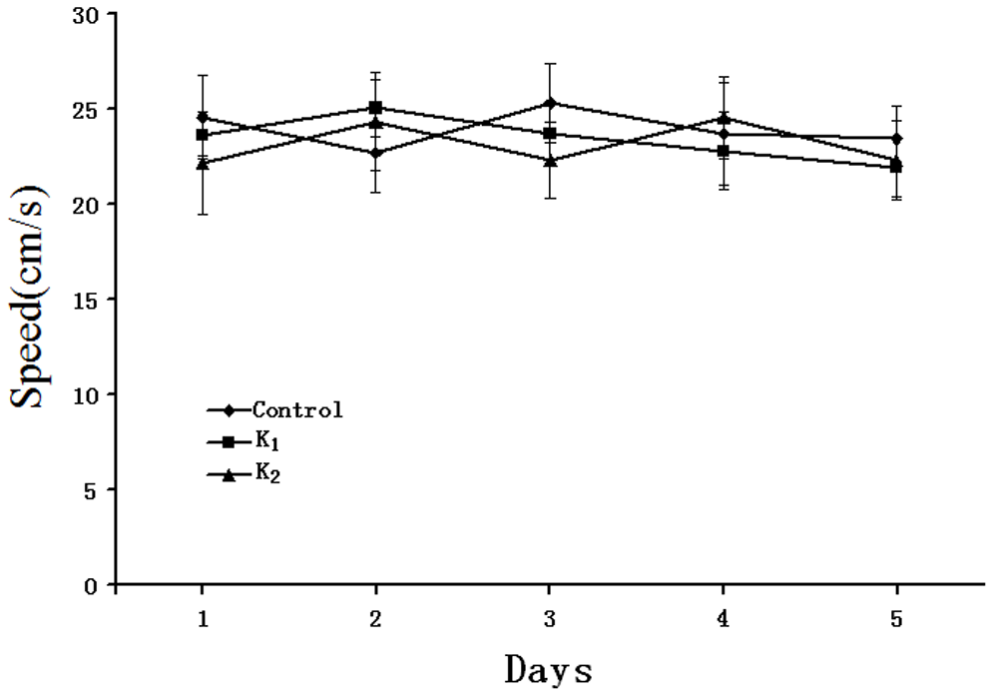
Mean swimming speed±S.E.M over the 5 days of testing in the Morris water maze. There was no difference between the three studied groups, or between days in each group.

There was no significant change in the mean swim speed between days during the study period in any of the three groups. Neither was there any difference in swim speed between the three groups (Fig. 2).

**Figure 3:**
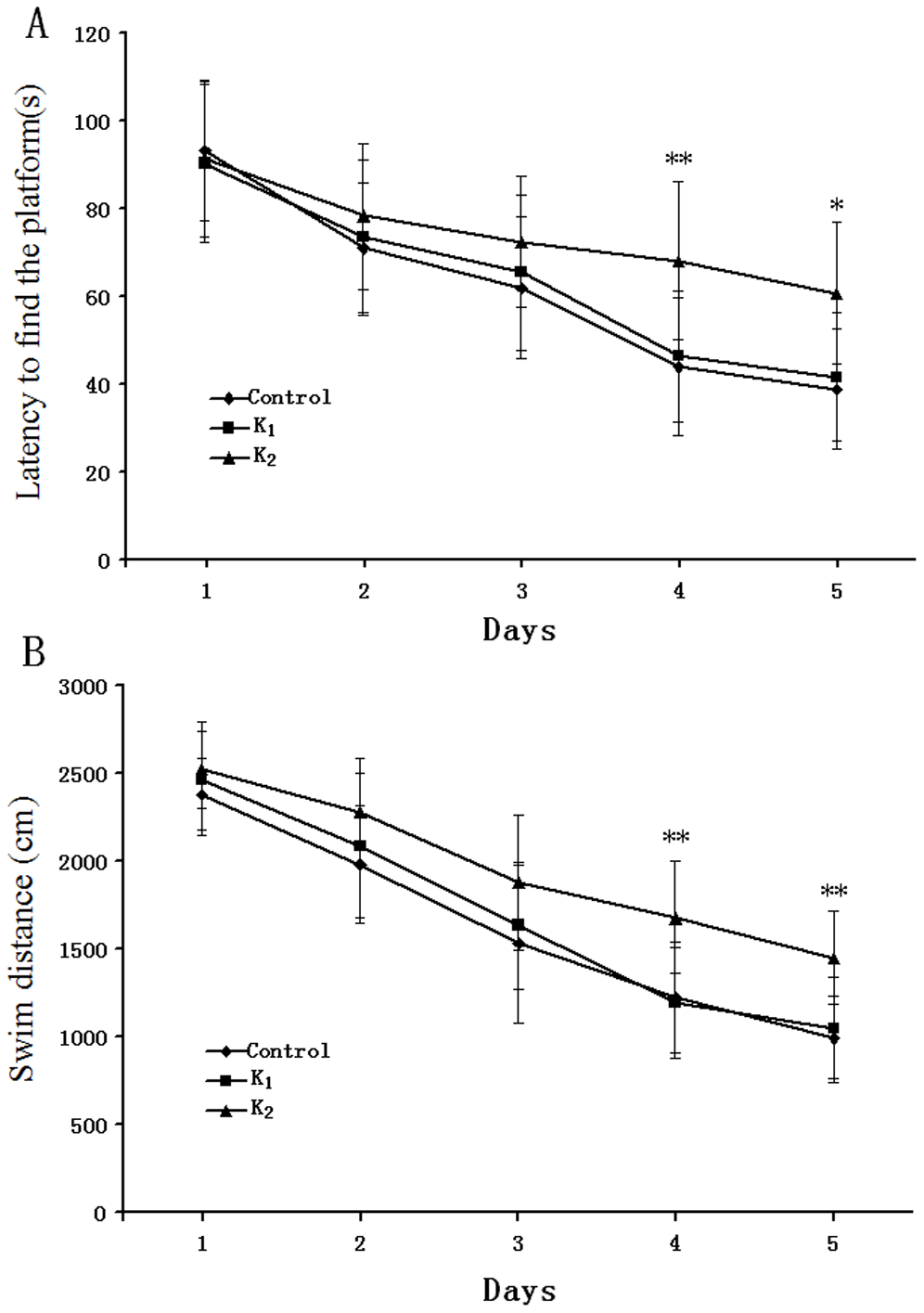
Effect of adolescent rat's exposure to different doses of ketamine on training trials in the Morris water maze. A: The latencies for finding the platform for each trial during the 5 day; B: Swim distance for finding the platform for each trial during the 5 day. Values are expressed as mean ± S.E.M, n = 10 for each group. *P<0.05, **P<0.01 vs Control on the fourth and fifth day(repeated-measures ANOVA, followed by Dunnett's test.

Twenty-four hours after the last treatment, the 30 rats were trained in the Morris water maze to test the learning and memory ability. The latency is shown in Fig. 3. As indicated in Fig. 3A, all animals showed a progressive decline in the escape latency with training, and the main effects of day [F_(4,108)_ = 89.308, p = 0.000] and group [F_(2,27)_ = 4.032, p<0.05] were significant. Further day analysis by Dunnett's test, we found that the rats in group K_2_ took longer to find the hidden platform on the fourth and fifth training days compared to the control(*p<0.05, **p<0.01). Furthermore, as indicated in Fig. 3B, the main effects of day [F_(2,27)_ = 156.931, p = 0.000] and group[F_(2,27)_ = 7.052, p<0.05] were also significant. Further day analysis showed that the rats in group K_2_ had an increased swimming distance to find the hidden platform on the fourth and fifth training days compared to the controls (**p<0.01).

**Figure 4:**
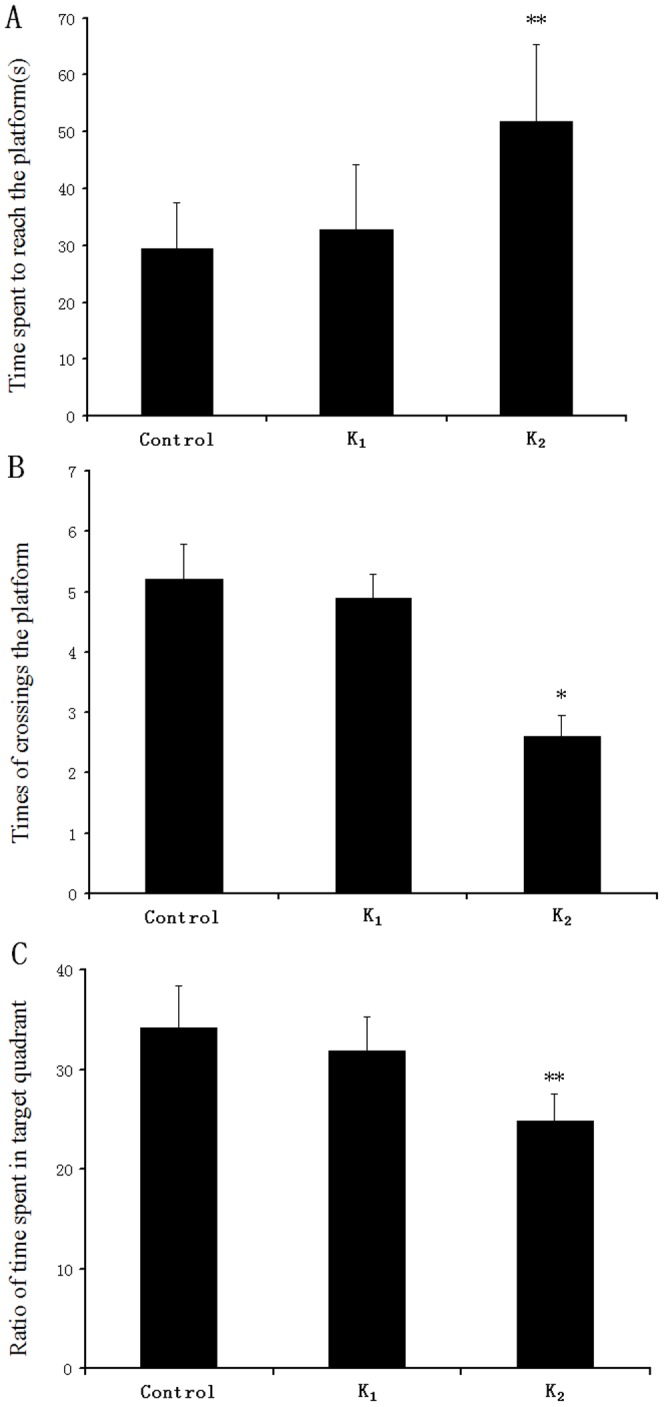
Effect of adolescent rat's exposure to different doses of ketamine on performance of spatial memory parameters in the Morris water maze. A:Time spent to reach the platform; B: Times of crossing the platform; C: Ratio of time spent in the target quadrant. Values are expressed as mean±S.E.M, n = 10 rats in for each group. Different groups compared to the Control, *p<0.05, **p<0.01 (one-way ANOVA, followed by Dunnett's test).

In the probe trial, the platform was removed, and the animals were placed in quadrant 2, which is opposite to the target quadrant (quadrant 4). The following endpoints were recorded: time spent to reach the platform initially, ratio of time spent in the target quadrant and the times of crossing the platform that the same animal crossed the former platform area. In the 120 s of the probe trial, there were significant differences in time spent to reach the platform initially, ratio of time spent in the target quadrant and the times of crossing the platform among all groups (F_(2,27)_ = 5.162, p<0.01; F_(2,27)_ = 4.802, p<0.05; F_(2,27)_ = 6.629, p<0.01, respectively). On further statistics by Dunnett's test, we found that groups K_2_ took longer to reach the platform initially than the controls(**p<0.01, Fig. 4A). The crossing times of group K_2_ were significantly fewer than the controls (*p<0.05, Fig. 4B). Furthermore, rats in the control group spent significantly more time in the target quadrant than those in group K_2_ (**p<0.01, Fig. 4C).

TUNEL staining of neurons at the hippocampal CA_1_ or cerebral cortex and subcortical regions

**Figure 5:**
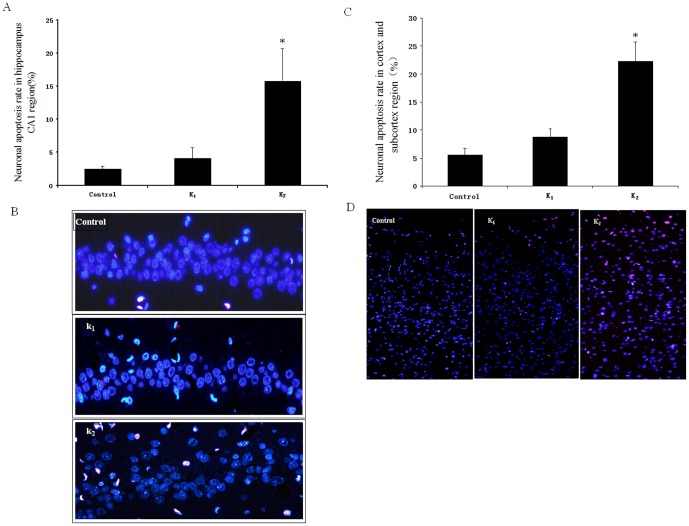
Neuronal apoptosis in rat hippocampal CA_1_ and cerebral cortex and subcortical region was measured by terminal deoxynucleotide transferase dUTP nick end labeling (TUNEL) staining. Quantitative analysis of neuronal apoptosis of hippocampal CA_1_ (A) and cerebral cortex and subcortical region (C) in Control, K_1_ and K_2_ group; Representative terminal deoxynucleotide transferase dUTP nick end labeling-stained photomicrographs of neurons (Red) of hippocampal CA_1_ (B) and cerebral cortex and subcortical region (D) in Control, K_1_ and K_2_ group. n = 6; *p<0.05 compared with Control group.

As shown in Fig. 5A–D, the apoptotic cells at CA_1_ or cerebral cortex and subcortical regions had a significant difference among the three groups. Further statistical analysis showed that the number of apoptotic cells in group K_2_ at CA_1_ (15.8%) or cerebral cortex and subcortex (22.3%) was significantly higher than that in Control (CA_1_:2.5%; cerebral cortex and subcortex:5.58%) or K_1_ group (CA_1_:4.1%; cerebral cortex and subcortex:8.78%) [F_CA1(2, 15)_ = 188.742, p<0.01; F _cerebral cortex and subcortex(2, 15)_ = 211.621, p<0.01].

### Immunohistochemistry of PTEN at the hippocampal CA_1_ or cerebral cortex and subcortical regions

**Figure 6:**
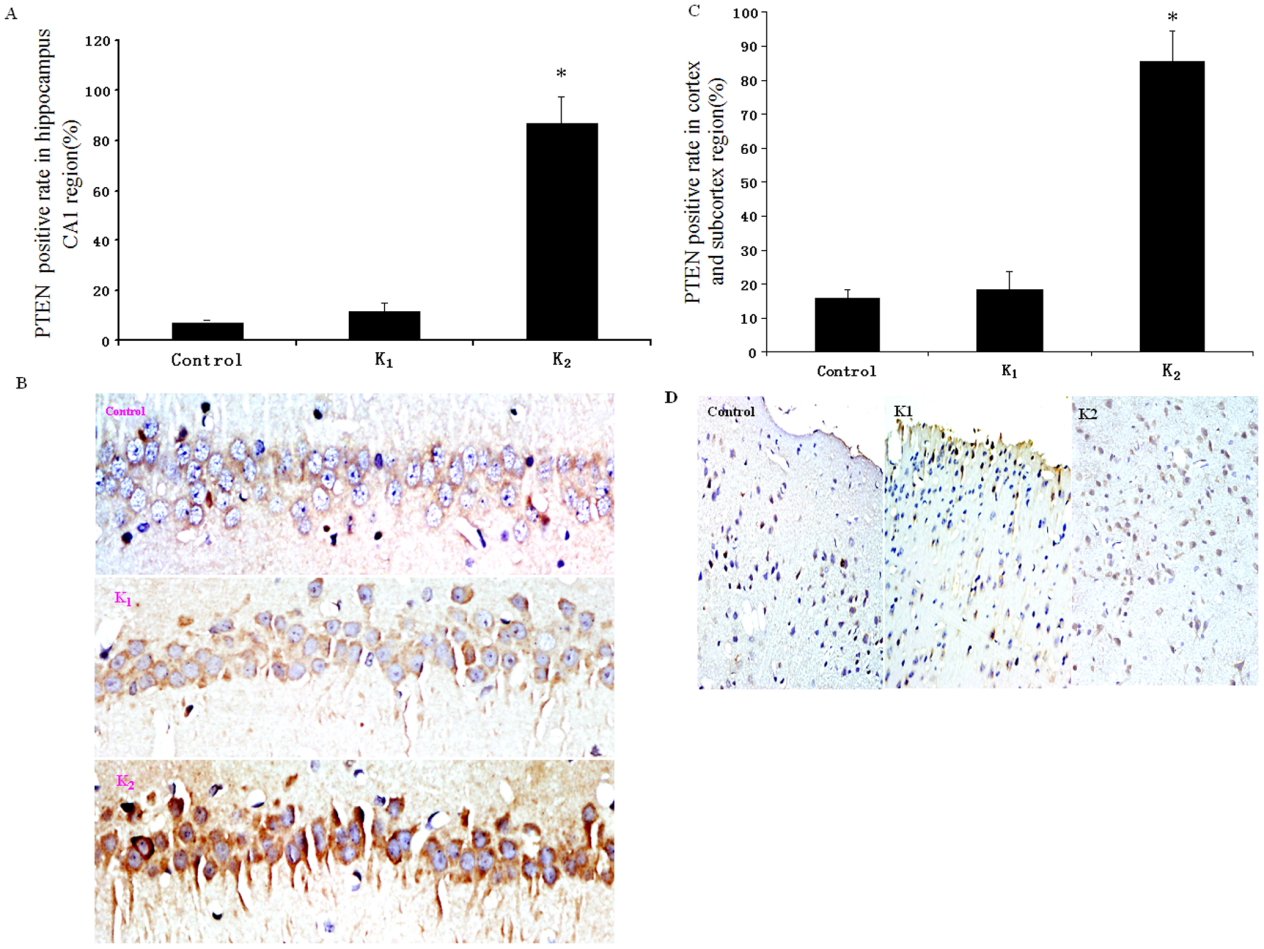
Neuronal PTEN expression in rat hippocampus CA_1_ and cerebral cortex and subcortical region were measured by immunohistochemistry analysis. Quantitative analysis of neuronal PTEN expression hippocampal CA_1_ (A) and cerebral cortex and subcortical region (C) in Control, K_1_ and K_2_ group. Representative PTEN expression positive neurons (yellow) of hippocampal CA_1_ (B) and cerebral cortex and subcortical region (D). n = 6; *p<0.05 compared with Control group.

As shown in Fig. 6A–D, the PTEN-expressed cells at CA_1_ or cerebral cortex and subcortical regions had a significant difference among the three groups. Further statistical analysis showed that the number of PTEN-positive cells in group K_2_ at CA_1_ (86.7%) or cerebral cortex and subcortex(85.4%) was significant higher than that in Control (CA_1_:6.7%; cerebral cortex and subcortex:15.8%) or K_1_ group (CA_1_:11.7%; cerebral cortex and subcortex:18.4%) [F_CA1(2, 15)_ = 215.828, p<0.01; F_cerebral cortex and subcortex(2, 15)_ = 176.581, p<0.01].

### qRT-PCR of miR-214 and western blot of PTEN protein

**Figure 7:**
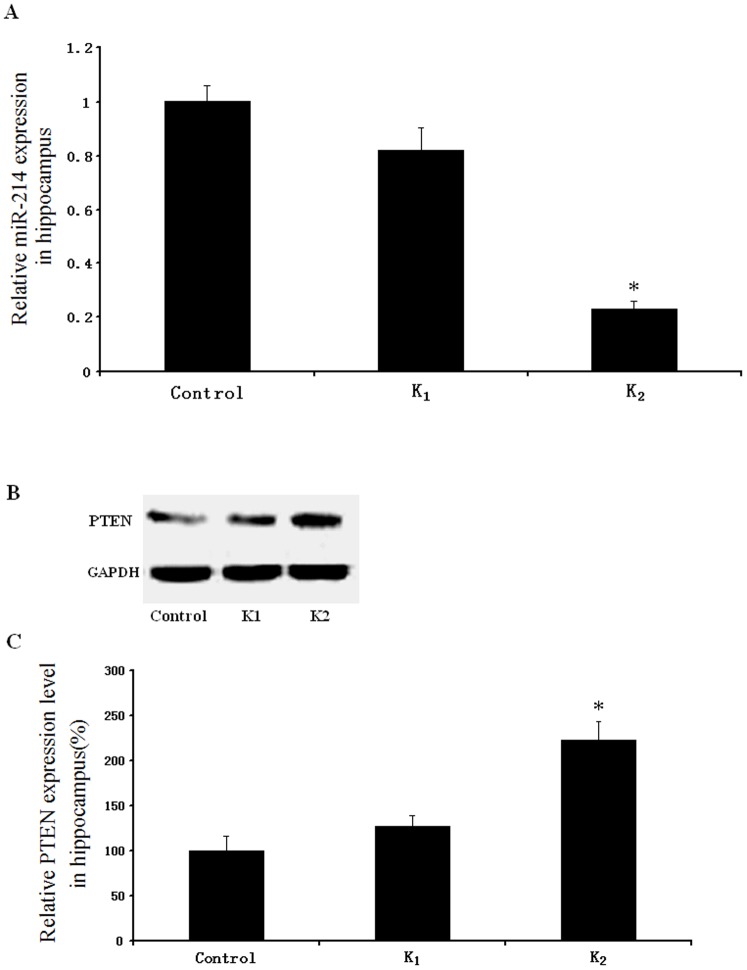
Relative miR-214 and PTEN protein expression levels in rat hippocampus were measured by qRT-PCR and western blot analysis. A: Quantitative analysis of hippocampus miR-214 levels in Control, K_1_ and K_2_ group, miR-214 expression were obviously decreased in K_2_ group rats (n = 6; *p<0.05 compared with Control group or K_1_ group). B: Representative western blots of hippocampus PTEN protein levels. C: Quantitative analysis of hippocampus PTEN levels in Control, K_1_ and K_2_ group, relative PTEN protein expression levels were obviously increased in K_2_ group rats (n = 6; *p<0.05 compared with Control group or K_1_ group). GAPDH, glyceraldehyde 3-phosphate dehydrogenase.

As shown in Fig. 7A, the expression of miR-214 at hippocampus had a significant difference among the three groups (p<0.05). Further statistical analysis showed that relative miR-214 level in group K_2_ was lower than that in Control or K_1_ group [F_(2, 15)_ = 250.734, p<0.01]. As expected, the expression of PTEN protein in group K_2_ was significantly higher than that in Control or K_1_ group [F_(2, 15)_ = 92.012, p<0.01] (Fig. 7B and 7C).

## Discussion

Ketamine, a noncompetitive NMDA receptor antagonist, produces a unique dissociative anesthetic state, which is particularly useful for inducing anesthesia in children for surgical procedures inside or outside the operating room [22]. In addition, both animal and clinical studies have demonstrated that ketamine also exerts a quick, and persistent antidepressant-like effect [2]–[7]. However, ketamine may also induce neurotoxicity in immature brains, although the molecular mechanisms responsible for side effect are still unclear [23], [24]. In current study, we aimed to determine the effects of ketamine on learning and memory function, and neuronal apoptosis of adolescent rats and to assess the potential effects of ketamine on the expression of miR-214 and PTEN in hippocampus.

Rats from 35–55 days old are adolescent or pubertal aged rats [25]. In this study, 90 SD rats (50-day-old), which are equivalent to 10–15 years old human subjects, were used. Therefore, the results from the current study might extend into adolescence. Based on previous reports from other groups [26]–[29], we applied ketamine at 30 and 80 mg/kg as the sub-anesthesic and anesthesic dose, and monitored the respiratory rate, heart rate, mean artery pressure and arterial blood gas at 5 min, 15 min, 1 h after 80 mg/kg ketamine administration in 5 rats in a pilot study. The pilot study showed that these parameters were within normal ranges (data not shown). In addition, the results of open-field test showed that 30 or 80 mg/kg ketamine (i.p, once per day for 7 consecutive days) had no significant effect on the spontaneous locomotor activity of rats.

As shown in the Morris water maze test [30], [31], we found that the repeated use of low dose of ketamine (30 mg/kg) did not obviously impair the learning and memory function of adolescent rats. Although studies from some groups have showed that ketamine administration in 30 mg/kg for 7 days can alter behavioral change in rats [32], [33], we think the methodology used may account for the difference. The above two papers employed social interaction test to focus on social interaction, whereas we applied Morris water maze test to evaluate the learning and memory function. However, the repeated use of high dose of ketamine (80 mg/kg) impaired learning and memory, including the escape latency and time or annulus crossings in the target quadrant. These behavioral disturbances suggest that the repeated exposure to the high dose of ketamine could impair the learning and memory function in the adolescent rats. The present findings underline the potential hazard of high doses of ketamine for neuroapoptosis and the decline of learning and memory function in adolescent rats.

We performed the experiment to determine whether or not the learning and memory impairment induced by the repeated use of high dose of ketamine is associated with neuronal apoptosis. The result demonstrated that the repeated use of high dose of ketamine could increase the neuronal apoptosis in adolescent rat. However, the pro-apoptotic effect of ketamine did not occur at low dose of ketamine. The deleterious effects of ketamine on the developing brain are consistent with some previous studies [34]–[36]. Hayashi and his colleagues also found that repeated administration of ketamine may lead to neuronal degeneration in the P7 rats [37].

Recent studies suggest that the high expression of PTEN could increase tumor cell apoptosis [16]–[20]. However, the effect of ketamine on the expression of PTEN in rat neurons is unclear. Our results from immunohistochemistry and western blot analysis showed that PTEN expression from rats with the repeated use of high-dose ketamine was much higher than that in control group and low-dose ketamine group. It is interesting that rats with higher PTEN expression had the increased apoptotic neuronal cells. These results suggest that neuronal apoptosis induced by ketamine may be related to the high expression of PTEN. It is reported that high PTEN expression in tumor cells increased tumor cell apoptosis, whether high PTEN expression would increase the neuronal apoptosis should be studied in future study. In addition, nearly all CA_1_ neurons being PTEN positive while there was only a modest increase in total PTEN protein levels from the western blot analysis. We think the difference may come from the different detection methods.

The mechanisms involved in ketamine-induced expression of PTEN are still unclear. Recent studies have shown that miRNAs could regulate mRNA translation and stability and could act as gatekeepers for many cellular genes. By promoting degradation of mRNAs or inhibiting their translation, miRNAs could serve as negative regulators for their target mRNAs [38], [39]. miR-214 was predicted to be capable of targeting PTEN based on bioinformatics analysis and other related researches [14], [15]. We therefore tested the effect of ketamine on the expression of miR-214 in the hippocampus of adolescent rats. The results showed that miR-214 expression level in high-dose ketamine group was lower than that in control or low-dose group.

It is well known that ketamine is often used acutely rather than chronically for inducing anesthesia in pediatric surgical procedures. The administration way (once a day for 7 consecutive days) in his study and our results might have no obvious guidance to pediatric ketamine anesthesia. However, the chronic administration of ketamine could be used for anti-depressive therapy.

In summary, in this study we have identified that chronic administration of high dose ketamine could induce the learning and memory impairment and increase the number of neuronal apoptotic cells of adolescent rats, in which the decreased miR-214 and increased PTEN expression might be involved.
